# Substandard and Falsified Medicines, Regulatory Capacity and Public Health: A Narrative Review With a Focus on Africa

**DOI:** 10.1002/puh2.70356

**Published:** 2026-08-31

**Authors:** Ahmed Vandy, Nancy Oluwakemi Samai, Onome Thomas Abiri, Michael Lahai, Fawzi Thomas, Tamba Buffa, Sheku Mansaray, Alvin Turay, Augustus Osborne

**Affiliations:** ^1^ Faculty of Pharmaceutical Sciences, College of Medicine and Allied Health Sciences University of Sierra Leone Freetown Sierra Leone; ^2^ Faculty of Medicine European University Tbilisi Georgia; ^3^ Institute for Development, Freetown Western Area Sierra Leone

**Keywords:** national medicine authorities, pharmacovigilance, public health medicine, regulatory capacity building, substandard and falsified medicines, WHO Global Benchmarking Tool

## Abstract

**Background:**

Weak medicines regulation contributes to the circulation of substandard and falsified medicines, undermining treatment outcomes, accelerating antimicrobial resistance and eroding public trust in health systems. This review synthesised evidence on regulatory structures and functions, mapped regional capacity, documented the public health consequences of regulatory failure and identified pathways toward stronger and more equitable pharmaceutical governance in Africa.

**Methods:**

This narrative review synthesised peer‐reviewed and grey literature published between 2000 and 2025. Sources were identified through PubMed/MEDLINE, Scopus, Web of Science, Google Scholar and institutional reports from the World Health Organisation (WHO), World Bank, African Union Development Agency New Partnership for Africa's Development (AUDA–NEPAD) and national regulatory authorities. Studies were eligible if they addressed regulatory functions, the consequences of substandard and falsified medicines, WHO Global Benchmarking Tool (GBT) maturity assessments, regional harmonisation initiatives or policy responses. The search yielded 633 records, of which 122 underwent full‑text review, and 38 met the inclusion criteria. Evidence was organised thematically across regulatory functions, capacity gaps, pathways of harm, documented incidents and regulatory strengthening initiatives.

**Results:**

Evidence shows that regulatory weaknesses affect public health through therapeutic failure, toxicity, antimicrobial resistance, avoidable expenditure and inequitable exposure among vulnerable populations. African regulatory systems show substantial heterogeneity, with some authorities achieving the WHO GBT Maturity Level 3, whereas many remain at lower maturity levels. Recent advances include digital regulatory platforms, reliance mechanisms, regional harmonisation, WHO prequalification and the establishment of the African Medicines Agency.

**Conclusion:**

Strengthening national regulatory authorities to WHO GBT Maturity Level 3 and above requires sustained political commitment, predictable financing, legal reform, robust post‑market surveillance, accredited laboratory capacity and effective regional reliance mechanisms. Medicines regulation should be recognised as a core public health and health‑security priority, serving as a foundational investment in protecting populations and ensuring equitable access to quality‑assured medicines.

## Graphical abstract

Weak medicines regulation in Africa enables the circulation of substandard and falsified medicines, undermining treatment outcomes, accelerating antimicrobial resistance and eroding public trust. This narrative review synthesised 38 studies from 2000 to 2025, highlighting regulatory gaps, public health harms and regional disparities. Although some authorities have achieved WHO GBT Maturity Level 3, many remain at Levels 1 and 2, with none at Level 4. Strengthening regulation requires political commitment, financing, accredited laboratories, surveillance, digitalisation and reliance mechanisms to ensure equitable access to quality‑assured medicines.

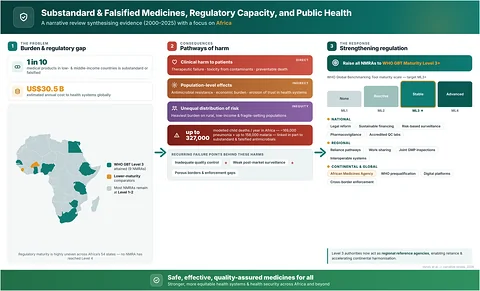

## 1 Introduction

Robust medicine regulation is a cornerstone of resilient health systems. In its absence, pharmaceutical supply chains are vulnerable to infiltration by unsafe, ineffective or poor‐quality products. Although the term ‘medical products’ encompasses medicines, vaccines, diagnostics, medical devices and blood products, the evidence synthesised in this review relates predominantly to medicines regulation; findings that extend to other product categories are indicated explicitly where relevant [1]. The World Health Organisation (WHO) estimates that one in ten medical products in low‐ and middle‐income countries (LMICs) is substandard or falsified, imposing an annual financial burden of approximately $30.5 billion on health systems globally [2]. Yet this figure understates the true cost: Preventable deaths from treatment failures, children harmed by contaminated syrups and communities losing trust in the medicines dispensed at their clinics [3, 4, 5, 6, 7]. These failures underscore that weak medicine regulation is not simply a technical gap but a profound public health threat.

The consequences of regulatory failure are neither abstract nor confined to low‐income settings. Documented incidents spanning Panama, the United States, China, Nigeria and Singapore demonstrate that harms arising from substandard and falsified medicines respect neither geographic nor economic boundaries [3, 7]. The capacity to detect and respond to pharmaceutical threats, however, remains deeply unequal between high‐income and lower income countries.

Although previous reviews have documented the prevalence and consequences of substandard and falsified medicines, fewer have integrated these harms with the regulatory system functions that could prevent, detect and respond to them. In particular, there is a need to connect the WHO Global Benchmarking Tool maturity (WHO GBT), African regulatory harmonisation, digital regulatory systems and public health outcomes within a single policy‐oriented synthesis.

The African continent presents a particularly relevant case study. A WHO assessment conducted in 2010 found that among 46 sub‐Saharan African countries, only 7% had moderately developed medicines regulatory systems, 63% demonstrated minimal regulatory capacity, and only 30% possessed a functioning national regulatory authority [8, 9]. Despite considerable progress since then, heterogeneity in regulatory maturity across the continent remains marked. This review examined the structure and function of medicines regulatory systems, mapped the regional landscape of regulatory capacity, documented the public health consequences of regulatory failure and identified evidence‐informed pathways toward stronger, more equitable pharmaceutical governance in Africa. By highlighting this evidence of regulatory challenges, this review offered solutions for strengthening medicines governance in Africa, reducing the burden of substandard and falsified products and guiding policy reforms that safeguard public health outcomes.

**Important definitions according to the** WHO [2, 10]:

- **Substandard medicines**: Authorised medicines that fail to meet either quality standards, specifications or both.
- **Falsified medicines**: Medicinal products that deliberately or fraudulently misrepresent their identity, composition or source.
- **Unregistered/unlicensed medicines**: Medicinal products that have not undergone evaluation and approval by the relevant national or regional regulatory authority.
- **Adverse drug reactions**: These are harmful and unintended effects of medicines that occur when the drugs are taken at the usual doses prescribed for treatment.

## 2 Methods

This study employed a narrative review design to examine the public health impact of weak medicine regulation in Africa. The focus was on regulatory system functions, capacity gaps, documented harms from substandard and falsified medicines and recent advances in regulatory strengthening. A narrative approach was chosen over a systematic review because of the breadth and heterogeneity of the evidence base, which includes surveillance reports, regulatory benchmarking assessments, case series, policy documents and grey literature. The study was not prospectively registered, consistent with narrative review methodology.

A structured literature search was conducted across PubMed/MEDLINE, Scopus, Web of Science and Google Scholar for publications between January 2000 and 2025, without emphasis on any specific period. Search terms combined keywords and Medical Subject Headings (MeSH) using Boolean operators. A representative PubMed search string was (‘substandard medicines’ OR ‘falsified medicines’ OR ‘counterfeit drugs’ OR ‘Counterfeit Drugs’) AND (‘Africa’ OR Africa OR ‘low‐resource settings’ OR ‘low‐ and middle‐income countries’) AND (‘medicines regulation’ OR ‘pharmaceutical regulation’ OR ‘national medicines regulatory authority’ OR ‘WHO Global Benchmarking Tool’ OR ‘African Medicines Agency’ OR ‘regulatory harmonisation’). Comparable strings were adapted for Scopus, Web of Science and Google Scholar. Reference lists of included studies were hand‐searched to identify additional sources.

Grey literature was reviewed, given its importance in regulatory policy. Sources included reports from the WHO, the World Bank, the African Union Development Agency New Partnership for Africa's Development (AUDA–NEPAD), the African Medicines Regulatory Harmonisation (AMRH) initiative and national regulatory authorities. The World Bank's 2020 consultancy report on medicines regulatory functions and policies across Africa was a primary source for country‐level data on National Medicines Regulatory Authority capacity and information management systems [11].

Sources were eligible if they addressed regulatory system functions, the prevalence or consequences of substandard and falsified medicines, WHO GBT maturity assessments, regional harmonisation initiatives in Africa or comparable LMICs or policy responses to regulatory gaps. Exclusion criteria included lack of full text, absence of substantive focus on medicines regulation, opinion pieces without original data and duplicate reporting. No language restriction was applied, though most sources were in English.

The combined database and grey‐literature search yielded 633 records. After removing duplicates and screening titles and abstracts, 122 records underwent full‐text review, of which 38 met eligibility criteria. Screening was conducted independently by three authors (A.V., N.O.S. and A.T.), with disagreements resolved by discussion with two authors (T.B. and S.M.). Grey literature was assessed for credibility on the basis of issuing organisation, transparency of methodology and recency. Greater weight was given to peer‐reviewed studies and internationally benchmarked reports.

Although a formal risk‐of‐bias assessment was not applied due to source heterogeneity, the review followed recognised good‐practice principles for narrative synthesis [12]. These included explicit eligibility criteria, transparent reporting of search methods and structured thematic organisation. Data were extracted using a structured framework and synthesised across five domains: essential regulatory functions and their public health impact; global and regional regulatory capacity; pathways of harm from weak regulation; documented case evidence; and advances and policy recommendations. Ethical approval was not required as only publicly available secondary data were used.

## 3 Essential Regulatory Functions and Their Public Health Impact

Medicine regulation encompasses a set of interdependent functions that together determine whether the pharmaceutical supply chain can be trusted to deliver safe, effective and quality‐assured products [13]. Regulatory failure is rarely the product of a single deficiency. It typically reflects simultaneous gaps across multiple domains, including inadequate post‐market surveillance, weak pharmacovigilance infrastructure, porous borders enabling illicit trade and the absence of harmonised regional standards [2, 13, 14, 15].

Marketing authorisation serves as the primary gateway through which medicines enter the legal supply chain [16]. By requiring pre‐market evidence of safety, efficacy and quality, regulatory authorities prevent the circulation of products that could cause direct harm or treatment failure. The integrity of this process depends on the scientific capacity of the reviewing agency and the rigour of its dossier evaluation procedures.

Licensing and inspection extend regulatory oversight beyond registration into the physical infrastructure of the pharmaceutical sector. Manufacturers, importers, distributors and pharmacy outlets must meet minimum standards before receiving authorisation to operate [17, 18]. Regular and risk‐based inspections verify ongoing compliance with Good Manufacturing Practices (GMP) and Good Distribution Practices (GDP), providing assurance that the standards applied at the point of registration continue to be met throughout a product's market lifecycle.

Pharmacovigilance is the science and activities relating to the detection, assessment, understanding and prevention of adverse drug reactions and other medicine‐related problems, an adverse drug reaction being any noxious and unintended response to a medicine [19]. It represents the regulatory system's post‐market immune response [20]. Once a medicine enters circulation, new safety signals may emerge from populations not represented in pre‐registration clinical trials, or from conditions of use that differ markedly from controlled trial settings [21]. A functional pharmacovigilance system collects, analyses and acts upon adverse drug reaction reports and quality complaints, detecting harm early before preventable deaths accumulate [20, 21]. Enforcement and quality control testing complete the regulatory architecture, providing the deterrent effect of legal sanction and the analytical foundation for evidence‐based decision‐making. Table 1 summarises the five core regulatory functions and their direct public health significance.

**TABLE 1:** Regulatory functions, scope and public health impact.

Regulatory function	Scope and activities	Public health impact
Marketing authorisation	Pre‐market scientific assessment of a medicine's safety, efficacy and quality before approval for sale	Prevents the circulation of unsafe, ineffective or poor‐quality products
Licensing and inspection	Registration and oversight of manufacturers, importers, distributors and pharmacies; routine and risk‐based inspections	Maintains supply chain integrity and ensures ongoing compliance with quality standards
Pharmacovigilance	Pre‐marketing safety monitoring during clinical development together with systematic post‐market monitoring for adverse drug reactions, adverse events following immunisation, product defects and safety signals	Enables early detection of harm, supports timely intervention and protects patients
Enforcement	Application of legal and administrative measures against regulatory violations, including product recalls and sanctions	Deters malpractice, removes harmful products and reinforces public trust in the health system
Quality control testing	Independent laboratory analysis to verify identity, strength, purity and stability	Detects substandard or falsified products, ensures therapeutic effectiveness and safeguards population health

## 4 Global and Regional Regulatory Landscape

### 4.1 The WHO GBT

The WHO GBT has become the internationally accepted standard for evaluating national regulatory system capacity [22]. Drawing on International Organization for Standardization (ISO 9004) quality management principles, the GBT classifies regulatory systems on a four‐level maturity scale [23]. Maturity Level 1 reflects no formal regulatory approach; Level 2 a reactive approach with some system elements in place; Level 3 a stable, formal system with most core functions performed effectively; and Level 4 continuous improvement aligned with international best practice [23, 24]. The tool assesses nine regulatory functions through more than 268 sub‐indicators, enabling both national self‐assessment and WHO‐conducted benchmarking exercises [25].

The GBT's significance extends beyond classification. Its results inform WHO‐listed authority designation, which enables reliance mechanisms through which one regulatory authority accepts and builds upon the decisions of another recognised authority [25]. This reliance pathway carries particular value in resource‐constrained settings, where conducting fully independent reviews of every product dossier is neither feasible nor necessary if a trusted authority has already evaluated the same product.

For Africa, the implications of the GBT framework are acute. As of 2024, 18 African countries had undergone official WHO‑conducted benchmarking, which involves external validation by WHO experts and may culminate in WHO‐listed authority status where thresholds are met. In contrast, 33 countries had completed self‑benchmarking, an internal exercise using the GBT tool without external validation [26]. These two processes are not equivalent and should not be conflated. Country‑level functionality assessments, such as those conducted by the World Bank or AUDA‑NEPAD, provide descriptive evaluations of regulatory operations but do not assign GBT maturity levels [26].

No African national regulatory authority had attained Maturity Level 4 at the time of this review. A growing number, including Tanzania, Ghana, Nigeria, Egypt, South Africa, Rwanda, Senegal, Zimbabwe and Ethiopia, have officially attained Level 3 for the specific product scopes and functions listed by WHO as of March 2026; for authorities not currently listed by WHO as ML3/ML4, this review avoids assigning a maturity level unless an official benchmarking report is cited [9, 27].

The majority of African National Medicines Regulatory Authorities (NMRAs), however, remain at Levels 1 or 2, characterised by limited enforcement capacity, partial pharmacovigilance systems and significant infrastructural constraints. Reliable published data on these countries are limited, as most Level 1/2 authorities undertake self‑benchmarking exercises that are not externally validated and therefore rarely disclosed. The few examples cited in Table 2 (e.g., Sierra Leone, Liberia, Côte d'Ivoire) are illustrative rather than exhaustive, drawn from available self‑benchmarking evidence. Table 2 provides detailed information on the WHO maturity levels, regulatory gaps and their implications for public health.

**TABLE 2:** World Health Organisation (WHO) medicines regulatory system maturity levels, key gaps and public health implications.

Country or region	GBT maturity level	Definition	Key regulatory gaps	Public health implications
Sierra Leone, Liberia, Côte d'Ivoire and majority of African countries	Levels 1 and 2	Elements of a regulatory system in place; consistent implementation limited	Weak enforcement, inadequate pharmacovigilance, limited post‐market surveillance	High risk of SF medicines entering the supply chain; delayed detection of safety issues
Egypt, Ethiopia, Tanzania, Ghana, Nigeria, Rwanda, Senegal, South Africa, Zimbabwe	Level 3	Most core regulatory functions performed effectively, with capacity for routine enforcement	Residual gaps in post‐market surveillance and cross‐border data sharing	Moderate SF risk; potential delays in regional safety alerts
No African country	Level 4	Regulatory system operates at international best practice with continuous improvement	Minimal systemic gaps; emphasis on innovation and quality enhancement	Low SF risk; strong capacity for rapid detection, recall and enforcement

Abbreviations: GBT = Global Benchmarking Tool; SF = substandard and falsified.

### 4.2 The African Regulatory Context

The African continent presents the most complex regulatory challenge globally. With 54 sovereign states, each governed by its own legislative framework, institutional architecture and resource base, the continental landscape is characterised by considerable diversity in both capacity and performance. The World Bank's 2020 consultancy for scoping a continental regulatory information management system documented regulatory functions across all African Union member states, revealing that although foundational functions, such as marketing authorisation and licensing, exist in most countries, operationalisation varies enormously [11].

Countries, such as Egypt, Tanzania, Ghana, Nigeria, Ethiopia, South Africa, Rwanda, Zimbabwe and Senegal, have made measurable and documented progress, evidenced specifically by their attainment of WHO GBT Maturity Level 3, the deployment of national digital regulatory information management systems, the establishment of functioning pharmacovigilance centres with active reporting to WHO's International Drug Monitoring Programme and formal participation in regional harmonisation mechanisms such as the AMRH and Africa Medicines Agency (AMA). Although all African countries except the Sahrawi Republic have a national regulatory authority or administrative unit performing some NMRA functions, most of their operations are limited or suboptimal [9, 28].

The World Bank (2020) data reveal that even among countries with formally established NMRAs, significant functional gaps persist. Angola had not registered any medicines at the time of the review owing to pending government approval. Libya's registration process was described as inactive, hampered by a lack of independence, minimal infrastructure and inadequately trained personnel. Lesotho had no legal provisions establishing the powers and responsibilities of its Medicines Regulatory Authority and no mandatory marketing authorisation requirement. These examples are illustrative of a broader pattern in which formal institutional existence does not reliably translate into functional regulatory capacity. The nine NMRAs profiled in Table 3 were purposively selected to represent regulatory and geographic diversity across the continent, comprising all African authorities officially listed by WHO at Maturity Level 3 at the time of writing together with two lower maturity authorities (Sierra Leone, Burkina Faso) included for comparative contrast; the table is illustrative rather than an exhaustive continental audit. See Table 3, which was compiled on the basis of information from the World Bank, WHO and AUDA–NEPAD reports [9, 11, 27, 29].

**TABLE 3:** Comparative overview of selected African National Medicines Regulatory Authorities (NMRAs): regulatory functionality and World Health Organisation (WHO) Global Benchmarking Tool (GBT) maturity.

Country	NMRA	Core functions	Regional integration	WHO GBT level (official year of attainment)	Key gaps and strengths
Egypt	Egyptian Drug Authority (EDA)	MA, PV, inspection, QC, ICT: all operational	AMRH (North Africa)	Level 3 (vaccines—2022) (medicines—2024)	Integrated digital RIMS; workforce retention remains a challenge
Rwanda	Rwanda Food and Drugs Authority (RFDA)	MA, PV, inspection, ICT: operational; QC: partial	EAC MRH/AMA	Level 3 (2024)	Reliance pathways well developed; financing sustainability is a concern
Ghana	Food and Drugs Authority (FDA Ghana)	MA, PV, inspection, QC, ICT: all operational	ECOWAS/AMA	Level 3 (medicines—2020) (vaccines [non‐producing]—2025)	Robust PV and track‐and‐trace system; regulatory science training gaps remain
Nigeria	National Agency for Food and Drug Administration and Control (NAFDAC)	MA, PV, inspection, QC: operational; ICT: partial	ECOWAS/AMA	Level 3 (Medicines—2022) (vaccines [non‐producing]—2025)	Digital PV reporting in place; fragmented state‐level enforcement
South Africa	South African Health Products Regulatory Authority (SAHPRA)	MA, PV, inspection, QC, ICT: all operational	SADC/AMA	Level 3 (Vaccines [producing]—2022)	Fully digitised systems; the historical 16,000‐application registration backlog inherited from the Medicines Control Council was cleared in 2022, though renewed application volumes continue to require active management
Tanzania	Tanzania Medicines and Medical Devices Authority (TMDA)	MA, PV, inspection, QC, ICT: all operational	EAC MRH/AMA	Level 3 (medicines—2018) (vaccines [non‐producing]—2025)	Digital GMP inspection and PV integration; funding stability required
Ethiopia	Ethiopian Food and Drug Administration (EFDA)	MA, PV, inspection, QC, ICT: all operational	IGAD/AMA	Level 3 (2025)	Early digital PV leadership; workforce retention challenges persist
Sierra Leone	Pharmacy Board of Sierra Leone (PBSL)	All functions: partial or developing	ECOWAS	Level 1 (2016)	AMRH roadmap under development; enforcement capacity limited
Burkina Faso	Directorate General of Pharmacy, Medicines and Laboratories (DGPML)	MA, inspection: operational; PV, QC, ICT: partial	ECOWAS	Level 1 (NA)	SIGIP ARP registration software in use; harmonised ICT tools lacking

Abbreviations: AMA = Africa Medicines Agency; AMRH = African Medicines Regulatory Harmonization; EAC MRH = East African Community Medicines Regulatory Harmonization; ECOWAS = Economic Community of West African States; GMP = Good Manufacturing Practice; ICT = Information and Communications Technology; IGAD = Intergovernmental Authority on Development; MA = marketing authorisation; NA = not available; PV = pharmacovigilance; QC = quality control; RIMS = Regulatory Information Management System; SADC = Southern African Development Community; SIGIP ARP = *Système Intégré de Gestion Informatisée des Processus Réglementaires* (Integrated System for Computerised Management of Regulatory Processes).

## 5 Pathways of Public Health Impact From Weak Medicines Regulation

**FIGURE 1:**
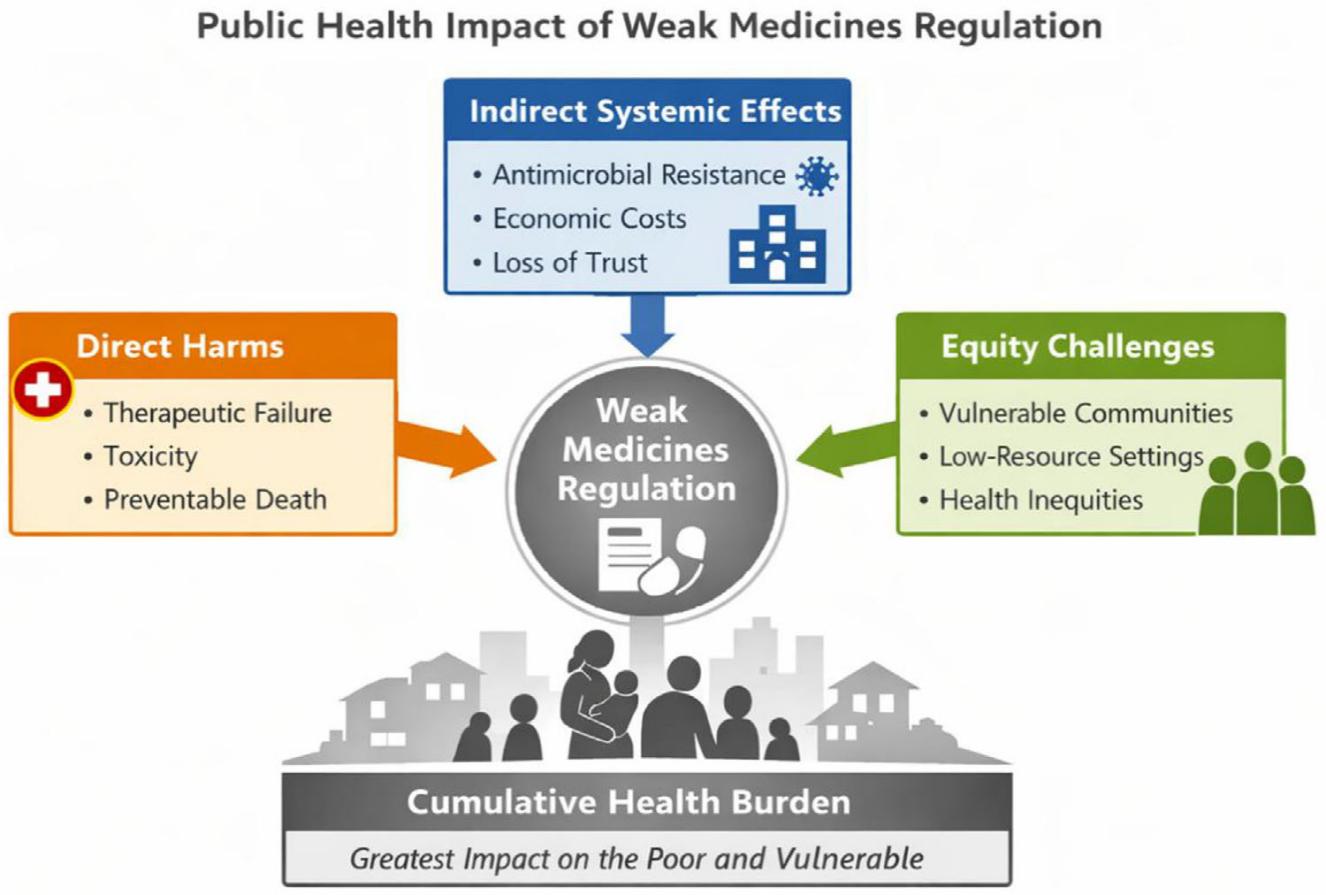
Schematic representation of the pathways through which weak medicines regulation affects public health. The figure illustrates three interconnected domains—direct harms (therapeutic failure, toxicity and preventable death), indirect systemic effects (antimicrobial resistance, economic burden and erosion of trust) and equity dimensions (disproportionate impact on vulnerable populations). Arrows indicate the cumulative progression of harm from individual to population level, culminating in heightened risk among the most marginalised. Evidence underlying the pathways depicted is drawn from sources [2, 32, 33, 36, 37, 38].

The public health consequences of regulatory failure operate through three distinct but interconnected pathways: direct harms to patients, indirect systemic effects at the population level and an inequitable distribution of risk that concentrates burden among the most vulnerable. These pathways are summarised in Figure 1, which depicts how weak medicines regulation produces direct clinical harms, amplifies systemic effects such as antimicrobial resistance and economic burden and disproportionately affects vulnerable populations.

### 5.1 Direct Harms: Therapeutic Failure, Adverse Effects and Preventable Death

The most immediately visible consequences of poor medicines regulation are the direct clinical harms experienced when patients receive substandard or falsified products [2, 4, 30].

Therapeutic failures due to substandard or falsified products occur when a medicine contains insufficient active pharmaceutical ingredient to achieve its intended effect. This was observed in cases of falsified phenobarbital in Guinea‐Bissau and Nigeria, which resulted in uncontrolled seizures and deaths among patients who believed they were receiving effective treatment [4]. Toxicity arises when products contain harmful contaminants or substituted substances, as seen in the diethylene glycol contamination events in Panama and Nigeria that collectively claimed hundreds of lives, the majority of them children [6].

These incidents are not isolated historical anomalies. The WHO Global Surveillance and Monitoring System continues to document cases of substandard and falsified medicines across multiple product categories and geographies. Almuzaini et al. conducted a systematic review of published case reports and identified incidents involving antimalarials, antibiotics, antihypertensives, anaesthetics and cancer therapies, demonstrating the breadth of therapeutic categories exposed to quality failure [31].

### 5.2 Indirect Effects: Antimicrobial Resistance, Economic Burden and Erosion of Trust

Regulatory failure extends beyond isolated clinical episodes to population‐level harms, most notably antimicrobial resistance. Substandard antibiotics containing sub‐therapeutic concentrations of active ingredients contribute to the emergence and propagation of resistant strains [32]. In malaria‐endemic regions, poor‐quality artemisinin‐based therapies have been documented as a contributing factor to resistant Plasmodium falciparum populations, threatening treatment efficacy across the continent [33].

The economic dimensions of regulatory failure are equally substantial, illustrating that both patients and health systems incur compounded costs when products that appear legitimate fail to deliver therapeutic benefit [34, 35]. Across Africa, modelled WHO estimates suggest that approximately 169,000 child deaths from pneumonia and up to 158,000 from malaria per year may be attributable, at least in part, to substandard and falsified antimicrobials; these figures derive from modelling exercises with inherent uncertainty rather than from direct case ascertainment and should be interpreted as indicative of scale rather than precise annual counts [36, 37]. These deaths represent not only human tragedy but also foregone economic productivity and cascading healthcare expenditure.

Regulatory failure also contributes to damaging the social contract between health systems and the populations they serve. When patients experience adverse reactions or therapeutic failures that may be attributable to poor‐quality medicines, the credibility of formal health services might be eroded [38]. Communities that lose confidence in official pharmaceutical supplies may seek treatment through informal markets or traditional remedies, further reducing the efficacy of public health interventions and compounding the original harm [37].

### 5.3 Equity Dimensions

The burden of regulatory failure is not distributed evenly. Vulnerable and underserved populations, including rural communities, low‐income households and patients in fragile state settings, face the greatest exposure to substandard and falsified medicines [2]. They have the fewest resources to seek alternative treatments when a product fails, the least access to quality‐assured pharmaceutical retailers and the most limited capacity to recognise and report adverse events [3]. These disparities are compounded by specific structural mechanisms: Rural populations often travel long distances to reach quality‐assured outlets, informal and unregulated drug markets fill gaps left by weak formal supply chains, high out‐of‐pocket expenditure pushes households toward cheaper and less‐regulated sources, complaint and adverse‐event reporting systems are least accessible where literacy and connectivity are lowest, procurement systems in fragile and conflict‐affected settings are frequently fragmented and difficult to monitor, and private pharmacies serving low‐income areas are less likely to be routinely inspected. Regulatory gaps thus compound existing health inequities, making regulatory strengthening an intrinsically equity‐relevant intervention and not merely a technical or administrative priority.

## 6 Documented Cases of Substandard and Falsified Medicines

The cases presented in Table 4 are drawn from published literature and synthesise incidents of substandard and falsified medicines across diverse product categories, regulatory contexts and health outcomes; they are not intended as an exhaustive inventory of all such incidents recorded over 2000–2025. Across all cases, common threads emerge: inadequate quality control of raw materials or finished products, insufficient post‐market surveillance and enforcement systems unable to deter actors operating outside the legitimate supply chain. These cases illustrate several recurring patterns. Contamination of raw materials during manufacture, falsification of finished products at the point of import or distribution and deliberate substitution of active ingredients are the most common mechanisms of harm. In each instance, a functioning regulatory system with robust quality control testing, effective import inspection and responsive post‐market surveillance would have created opportunities for early detection and prevention. The concentration of the most severe incidents in lower income settings reflects both the greater prevalence of regulatory gaps and the heightened vulnerability of populations with fewer alternative sources of quality‐assured healthcare.

**TABLE 4:** Documented cases of substandard and falsified medicines: causes, health impacts, regulatory gaps and responses.

Country and year	Cause	Health impact	Regulatory gap	Response or proposed response	References
The Gambia, 2022	Cough syrups contaminated with diethylene glycol and ethylene glycol	Outbreak of acute kidney injury with case fatality rates >80%	Weak import testing, limited pharmacovigilance, reliance on unverified overseas suppliers	Strengthen laboratory capacity; enforce WHO prequalification; establish cross‐border accountability mechanisms	[39, 40, 41]
Nigeria, 2008	Paracetamol teething mixture with diethylene glycol	Lead to the death of at least 84 children	Inadequate post‐market surveillance; poor enforcement against unregistered products	Ban open drug markets; enforce licensing of all manufacturers and distributors	[42, 43]
Guinea‐Bissau, 2013	Falsified phenobarbital	74 of 117 patients with seizure reported recurrence; 2 deaths	Absence of routine quality testing for essential medicines	Establish national quality control laboratories; pursue regional pooled testing arrangements	[44, 45]
Congo, 2014	Falsified diazepam containing haloperidol	930 dystonic reactions; 11 deaths	No pre‐distribution quality verification; poor market surveillance	Implement random batch testing; enforce penalties for falsification	[46]
Africa, multiple countries	Administration of substandard and falsified antimicrobials	Approximately 169,000 child deaths annually from pneumonia; up to 158,000 from malaria	Weak regulatory capacity; porous borders; limited pharmacovigilance	Regional harmonisation through the African Medicines Agency; WHO GBT capacity building; cross‐border enforcement	[37]

## 7 Recent Advances in Regulatory Strengthening

The picture of regulatory capacity in Africa and other lower income regions is not static. Significant institutional progress has occurred in recent years, driven by political will, international support and growing recognition that regulatory investment yields tangible and measurable returns for health and economic development.

### 7.1 WHO GBT Maturity Level 3 Attainment by African NMRAs

The achievement of WHO GBT Maturity Level 3 by the African NMRAs identified in Tables 2 and 3 represents a landmark development, reflecting sustained investment in workforce training, legal reform, digital infrastructure and regional collaboration. Beyond their domestic significance, Level 3 authorities are now positioned to serve as regulatory reference agencies for neighbouring countries, enabling reliance mechanisms and accelerating harmonisation across the continent. The African Medicines Agency itself, established by treaty in 2019 and in force since 2021, was operationalised in 2025 with the appointment of its Director‐General and remains in its foundational implementation phase in 2026, with universal ratification and financial self‐reliance targeted by 2030 [27, 47].

### 7.2 The African Medicines Agency

The establishment of the African Medicines Agency represents the most significant structural development in African medicines regulation in recent decades. The AMA Treaty was adopted by the African Union in February 2019 and entered into force in November 2021 following its 15th ratification; its Secretariat is headquartered in Kigali, Rwanda, and became operational following the appointment of its inaugural Director‐General in 2025 [47, 48]. As of early 2026, 32 of the African Union's 55 member states had ratified the Treaty, and full continental implementation remains contingent on universal ratification, domestication into national law and sustainable financing. Mandated to coordinate regulatory harmonisation, improve cross‐border enforcement and build capacity across the continent's NMRAs, the AMA is positioned to transform the regulatory landscape by reducing duplication, optimising scarce expertise and enabling faster access to quality‐assured medicines [48]. Joint product reviews, shared GMP inspection programmes and coordinated market surveillance are among the functions the AMA is designed to deliver at continental scale; as of 2026 these remain in early or pilot implementation among ratifying states rather than fully operational continent‐wide, and their scale‐up depends on the pace of ratification and financing.

### 7.3 Digital Regulatory Platforms and Information Systems

The adoption of digital regulatory information management systems has materially improved the efficiency and transparency of regulatory processes in several African countries. Tanzania's Integrated Regulatory Information Management System encompasses online product registration, GMP inspection management and a clinical trials registry, bringing coherence to functions previously managed through disparate paper‐based processes. Nigeria's NAFDAC Automated Product Administration and Monitoring System (NAPAMS) platform enables electronic product registration, Rwanda has implemented online import permit processing through its Product Regulatory Information Management System (PRIMS), and Ghana's Safety Watch System provides an International Council for Harmonisation (ICH) E2B‐compliant database for pharmacovigilance reporting, enabling direct submission of adverse drug reaction data to the WHO International Drug Monitoring Programme. The deployment of VigiMobile for pharmacovigilance by the Uppsala Monitoring Centre across several countries, like Zimbabwe, Sierra Leone and other African countries, is also a game changer for reporting of adverse drug reactions, adverse events following immunisation and other medicine‐related problems [49].

These platforms reduce processing time, improve data accessibility and create audit trails that support institutional accountability. They also facilitate the data‐sharing arrangements that regional harmonisation requires. The World Bank documented, however, that many countries continue to rely on paper‐based systems or isolated databases that cannot readily interface with regional platforms, underscoring the continued need for investment in interoperable digital infrastructure [11].

### 7.4 WHO Prequalification and Capacity Building

The WHO Prequalification Programme, expanded to include active pharmaceutical ingredients, diagnostics and finished pharmaceutical products, provides a globally recognised quality benchmark that procurement agencies and regulatory authorities can rely upon [50]. Countries participating in prequalification processes benefit from technology transfer, training and the development of internal expertise, which strengthen broader regulatory functions. Complementary capacity‐building initiatives, including WHO GBT‐linked training programmes, regional centres of regulatory excellence established through the African Medicines Regulatory Harmonisation initiative and donor‐funded technical assistance, have collectively contributed to measurable improvements in regulatory capacity across the continent.

## 8 Policy Recommendations for Strengthening Medicines Regulation

**FIGURE 2:**
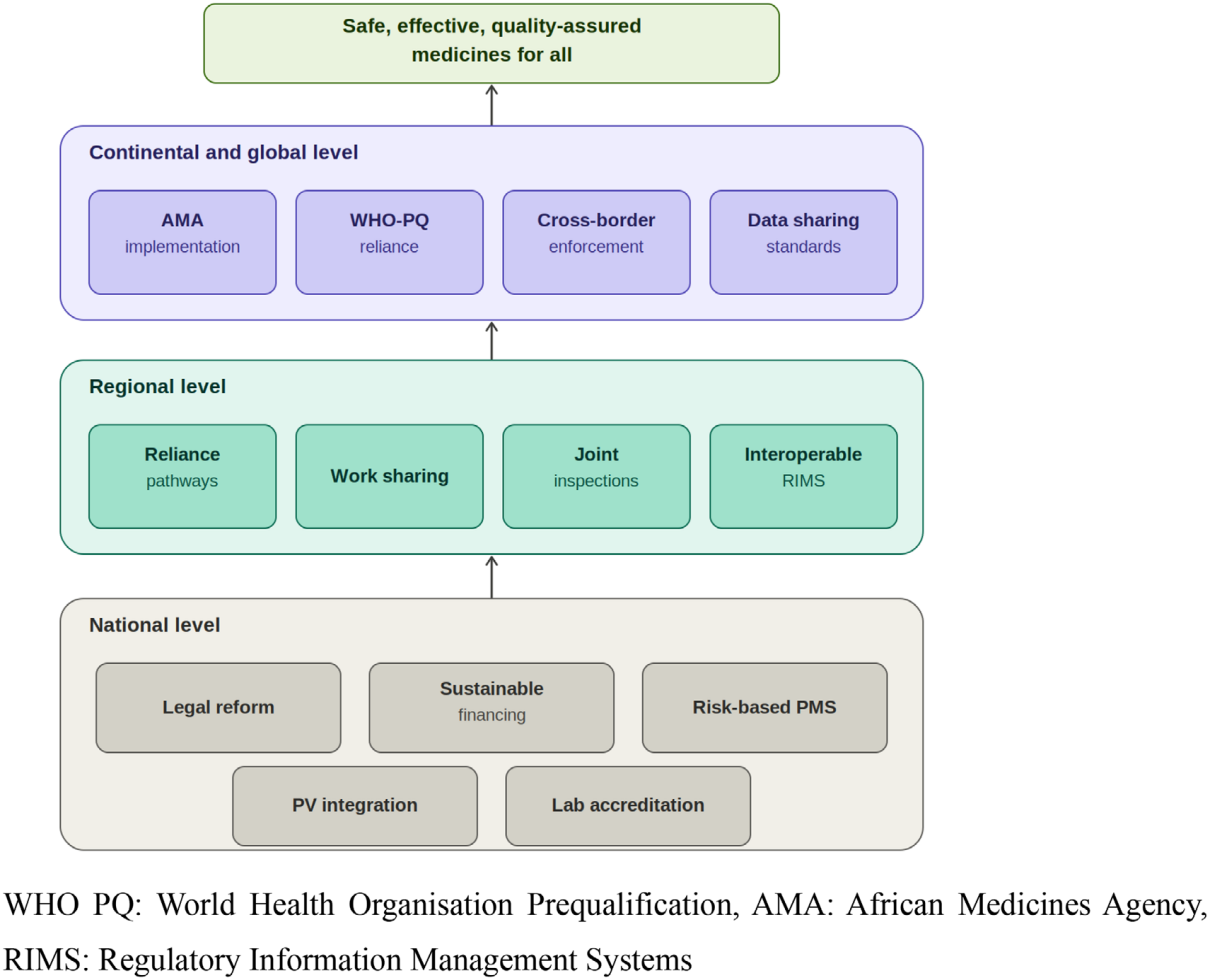
Policy pathways for strengthening medicines regulation, organised into national, regional and continental/global levels, converging toward safe, effective, quality‐assured medicines for all. AMA, African Medicines Agency; RIMS, regulatory information management systems; WHO PQ, World Health Organisation Prequalification.

The evidence reviewed supports policy action at three levels: national, regional and continental or global, reflecting the different actors responsible for implementation. Figure 2 summarises how these levels interact to advance regulatory maturity and equitable access to quality‐assured medicines.

### 8.1 National Level

#### 8.1.1 Legal Reform

National laws should align with the African Union Model Law on Medical Products Regulation, giving authorities clear power to recall products, seize markets and prosecute offenders.

#### 8.1.2 Sustainable Financing

GBT‐informed institutional development plans should be costed into national health budgets, not left dependent on donor cycles.

#### 8.1.3 Risk‐Based Surveillance

Post‐market surveillance should target the highest risk therapeutic areas first, such as malaria, tuberculosis and HIV medicines.

#### 8.1.4 Pharmacovigilance Integration

Adverse drug reaction data and quality complaints should feed a single regulatory response system, with public dashboards for transparency.

#### 8.1.5 Laboratory Accreditation

WHO‐prequalified and ISO/IEC 17025‐accredited quality control laboratories are the analytical backbone of credible enforcement.

### 8.2 Regional Level

#### 8.2.1 Reliance Pathways

Lower maturity NMRAs should formally rely on assessments already conducted by Level 3 authorities, cutting duplication and speeding access.

#### 8.2.2 Work Sharing

Joint dossier reviews across NMRAs spread technical workload and build assessor skills simultaneously.

#### 8.2.3 Joint Inspections

Coordinated GMP inspections reduce manufacturer burden and extend reach into countries with limited inspectorate capacity.

#### 8.2.4 Interoperable Systems

Standardised regulatory information platforms are the technical precondition for reliance and work sharing to function at scale.

### 8.3 Continental and Global Level

#### 8.3.1 AMA Implementation

Full operationalisation of joint reviews, shared inspections and coordinated trial oversight depends on continued treaty ratification and sustainable financing.

#### 8.3.2 WHO Prequalification (WHO‐PQ) Reliance

Wider use of WHO‐PQ outcomes for accelerated registration lets countries benefit from an international benchmark while building internal expertise.

#### 8.3.3 Cross‐Border Enforcement

Shared customs intelligence, joint investigations and harmonised sanctions are needed to disrupt supply chains that exploit fragmented borders.

#### 8.3.4 Data‐Sharing Standards

Common technical standards for regulatory data exchange underpin every other continental initiative and merit dedicated investment.

Embedding these actions within universal health coverage roadmaps and Sustainable Development Goal frameworks, alongside public–private partnerships and community‐level reporting, keeps regulatory strengthening measured, resourced and politically prioritised.

## 9 Conclusion

Weak medicines regulation remains a silent but powerful driver of preventable illness, death and economic loss. The evidence reviewed in this article demonstrates that substandard and falsified medicines undermine treatment outcomes across therapeutic categories, accelerate antimicrobial resistance, erode public trust in health systems and impose the heaviest burdens on the most vulnerable populations. The global pharmaceutical supply chain's increasing complexity, with raw materials and finished products traversing multiple jurisdictions before reaching patients, means that regulatory failures in one country generate harms in many others.

Africa faces particular urgency. Most of the continent's NMRAs remain below WHO GBT Maturity Level 3, and the health consequences of this regulatory deficit are visible in child deaths, treatment failures and the persistence of substandard antimicrobials in markets where they are most needed. The evidence is equally clear that progress is both possible and verifiable. Countries that have invested in their regulatory systems, pursued legal reform, built technical capacity, adopted digital tools and engaged constructively with regional harmonisation have achieved Level 3 and are delivering measurable public health benefit.

Achieving WHO Maturity Level 3 and above across all National Medicines Regulatory Authorities demands sustained political commitment, adequate and predictable financing for regulatory institutions, technical capacity building that keeps pace with an evolving pharmaceutical landscape and coordinated regional and global action through the African Medicines Agency, WHO and allied international partners. Medicines regulation is not a marginal technical undertaking. It is a foundational investment in the health security of populations across Africa and the world.

## Author Contributions

Ahmed Vandy, Nancy Oluwakemi Samai and Michael Lahai conceptualised and designed the study. Ahmed Vandy, Alvin Turay, Nancy Oluwakemi Samai, Tamba Buffa and Sheku Mansaray conducted the literature review and data curation. Ahmed Vandy, Nancy Oluwakemi Samai and Augustus Osborne wrote the first draft of the manuscript. Ahmed Vandy, Augustus Osborne, Tamba Buffa and Fawzi Thomas critically revised the manuscript for important intellectual content. Onome Thomas Abiri and Michael Lahai supervised the study. All authors have read and approved the final manuscript.

## Funding

The authors have nothing to report.

## Ethics Statement

The authors have nothing to report.

## Consent

The authors have nothing to report.

## Conflicts of Interest

The authors declare no conflicts of interest.

## Data Availability

Not applicable because no new data or databases were used in the preparation of this work.
